# Polycystic Ovarian Morphology and Chronic Morbidity and Mortality in PCOS

**DOI:** 10.1001/jamanetworkopen.2025.40818

**Published:** 2025-10-31

**Authors:** Nir Kugelman, David V. Morris, Marie-Pier Bastrash, Tehila Feinberg, Sofia Miconiatis, Jordanna Rehany, Seang Lin Tan, Michael H. Dahan

**Affiliations:** 1 Division of Reproductive Endocrinology and Infertility, Department of Obstetrics and Gynecology, McGill University, Montreal, Quebec, Canada; 2 Rappaport Faculty of Medicine, Technion-Israel Institute of Technology, Haifa, Israel; 3 Department of Endocrinology, McGill University, Montreal, Quebec, Canada; 4 Department of Obstetrics and Gynecology, McGill University, Montreal, Quebec, Canada; 5 Medical School, McGill University, Montreal, Quebec, Canada; 6 Department of Pathology, McGill University, Montreal, Quebec, Canada; 7 Department of Nursing, McGill University, Montreal, Quebec, Canada

## Abstract

**Importance:**

Polycystic ovary syndrome (PCOS) is a prevalent endocrine disorder associated with insulin resistance and cardiovascular risk. The long-term association of ovarian morphology subtypes with long-term morbidity and mortality remains unclear.

**Objective:**

To determine whether long-term morbidity and mortality differ among women with PCOS with or without polycystic ovarian morphology (PCOM).

**Design, Setting, and Participants:**

This prospective cohort study was conducted from 1987 to 2005 with follow-up until 2024 (37 years) at a single academic referral center where women with PCOS consented for long-term health monitoring. Of 1089 initially enrolled women, 340 women with PCOS and sonographic data at baseline were included. Data were analyzed from January to April 2024.

**Exposure:**

Ovarian morphology was defined by the presence (PCOM) or absence (non-PCOM) of 12 or more peripherally distributed 2- to 9-mm follicles.

**Main Outcomes and Measures:**

All-cause mortality and chronic morbidity, including diabetes and cardiovascular, neurologic, thyroid, respiratory, gastrointestinal, kidney, autoimmune, psychiatric, and cancer conditions were assessed. Demographics, hormone levels, and cardiovascular risk factors were collected at enrollment. Outcomes were assessed at follow-up in older age, with data analyzed using χ^2^ tests, *t* tests, and multivariate logistic regression adjusted for confounders (baseline age, body mass index, fasting insulin, lipids, and follow-up duration).

**Results:**

Among 340 women with PCOS, 189 women had PCOM (mean [SD] age at enrollment, 28.03 [5.98] years) and 151 women did not (mean [SD] age at enrollment, 32.57 [9.21] years). Women with PCOM were younger at enrollment (*P* < .001), with higher mean (SD) body mass index (27.20 [5.74] vs 25.31 [6.31]; *P* = .004), cholesterol ratios (70.64 [51.76] vs 48.36 [51.84]; *P* < .001), and levels of luteinizing hormone (7.28 [8.60] mIU/mL vs 3.47 [4.39] mIU/mL; *P* < .001), androgens (eg, total testosterone: 90.78 [37.18] ng/dL vs 52.74 [76.66] ng/dL; *P* < .001), fasting insulin (12.84 [9.83] μIU/mL vs 8.85 [6.33] μIU/mL; *P* < .001), total cholesterol (167.57 [63.32] mg/dL vs 147.1 [82.24] mg/dL; *P* = .01), and triglycerides (97.35 [72.57] mg/dL vs 72.57 [61.06] mg/dL; *P* < .001). Mean (SD) follow-up duration was shorter in women with PCOM (33.7 [2.3] years vs 35.1 [4.9] years; *P* < .001). Mortality rates were similar, although mean (SD) age at death was younger in women with PCOM (54.3 [11.5] years vs 70.8 [13.6] years; *P* = .08). Non–insulin-dependent diabetes was significantly more common in women with PCOM (45 women [23.8%] vs 14 women [9.3%]; *P* < .001). Hypertension was not statistically significantly different in women with PCOM. Multivariate analysis confirmed the association of PCOM with non–insulin-dependent diabetes (aOR, 2.92; 95% CI, 1.06-2.82; *P* = .02). No other significant differences in chronic diseases were observed.

**Conclusions and Relevance:**

In this study, the PCOM pattern was associated with early metabolic abnormalities and increased risk of type 2 diabetes but not with increased mortality or other chronic diseases.

## Key Points

**Question:**

Is ovarian morphology in women with polycystic ovary syndrome (PCOS) associated with long-term morbidity and mortality?

**Findings:**

In this cohort study with 37 years of prospective follow-up of 340 women with PCOS, those with polycystic ovarian morphology (PCOM) had significantly higher rates of non–insulin-dependent diabetes (23.8% vs 9.3%) than those without this pattern. No significant differences were found in overall mortality or other chronic diseases.

**Meaning:**

This study found that PCOM in PCOS was associated with increased long-term risk for type 2 diabetes but not with increased mortality or broader chronic disease burden.

## Teaser

This cohort study investigates the association of polycystic ovarian morphology with long-term morbidity and mortality in women with polycystic ovary syndrome (PCOS).

## Introduction

Polycystic ovary syndrome (PCOS) is a common endocrine disorder affecting approximately 6% to 22% of women of reproductive age, characterized by hyperandrogenism, menstrual irregularities, and polycystic ovarian morphology (PCOM).^1^ Women with PCOS are at increased risk for metabolic and cardiovascular conditions,^2^ including insulin resistance, dyslipidemia, and hypertension, which are components of the metabolic syndrome. This syndrome is associated with an increased risk of heart disease, stroke, and type 2 diabetes, with a greater prevalence among women with PCOS than in the general population.^3,4,5^

Despite extensive research, the long-term morbidity and mortality associated with PCOS, particularly regarding ovarian morphology, are not fully understood.^6^ Many prior studies were limited by short follow-up durations and small sample sizes, restricting their ability to detect long-term health outcomes.^7^

One diagnostic criterion for PCOS is PCOM, historically referred to as the “string of pearls” appearance on ultrasonography, defined by 12 or more small follicles (2-9 mm) arranged peripherally in the ovary on transvaginal ultrasonography.^1^ Increased ovarian stromal volume, including theca cells, contributes to excessive androgen production and associated metabolic disturbances.^8,9,10^ Studies have reported that women with PCOM exhibit more severe metabolic disturbances, including higher levels of insulin resistance, hyperandrogenism, and elevated body mass index (BMI; calculated as weight in kilograms divided by height in meters squared) compared with women without PCOM.^9,11^ However, the long-term association of PCOM with chronic morbidity and mortality has not been definitively established.^12^

This study aimed to address these limitations by analyzing long-term morbidity and mortality in women with PCOS with and without PCOM over a 37-year follow-up. This study sought to determine whether ovarian morphology was associated with long-term health outcomes.

## Methods

### Study Design and Participants

This prospective cohort study was conducted at the McGill University Endocrinology Clinic. Women were enrolled between 1987 and 2005 and followed up until 2024. All participants provided informed consent for long-term health tracking. The study was approved in 1984 by the Department of Obstetrics and Gynecology ethics board of the Royal Victoria Hospital prior to the creation of the McGill Hospital Institutional Review Board. This approval granted long-term follow-up of the women (D.V.M.). This study is reported following the Strengthening the Reporting of Observational Studies in Epidemiology (STROBE) reporting guideline.

A total of 1089 women were recruited from the same academic clinical setting. Documentation on the number of women approached but not enrolled is unavailable. Of women enrolled, 195 women (17.9%) were lost to follow-up, leaving 894 women for analysis, including 720 women with PCOS and 174 women in the ovulatory control group. For this analysis, we included only the 340 women with PCOS who had baseline transvaginal sonographic data. Of these, 189 women had a PCOM ovarian appearance and 151 women did not (Figure). Race and ethnicity were not collected as part of the original study protocol given that these data were not routinely documented at our institution during the enrollment period.

**Figure.**
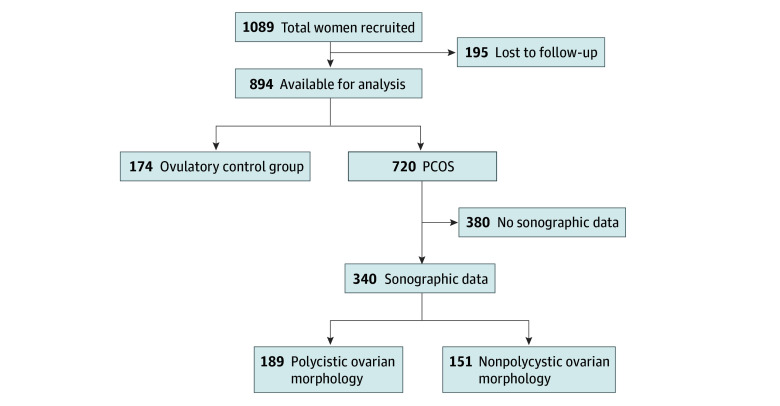
Study Flowchart PCOS indicates polycystic ovary syndrome.

### Inclusion and Exclusion Criteria

Women were eligible for inclusion if they were aged 18 years or older and diagnosed with PCOS based on the presence of both oligomenorrhea or amenorrhea and clinical or biochemical signs of hyperandrogenism. Other causes of these symptoms, such as congenital adrenal hyperplasia, hypothyroidism, hyperprolactinemia, and androgen-secreting tumors, were excluded.

### Study Groups

At enrollment, participants were divided into a study group and a control group. The study group included women diagnosed with PCOS based on the criteria described previously. The control group comprised ovulating women without clinical or biochemical hyperandrogenism; ovulatory status was defined by regular menstrual cycles. Women in the control group were not matched and were excluded from this analysis, which focused solely on women with PCOS.

For this analysis, only women with PCOS who had available transvaginal sonographic information were included. These selected women were divided into 2 groups based on their ovarian morphology as determined by ultrasonography: (1) The PCOM group was defined by the presence of 12 or more small follicles (2-9 mm in diameter) arranged peripherally in at least 1 ovary involving at least 50% of the ovarian circumference, as measured in the central cross-section of the ovary on transvaginal ultrasonography. (2) The non-PCOM group included participants without this peripheral pattern, in whom follicles were dispersed throughout the stroma in the central cross-section. Note that although this classification was historically referred to as the “string of pearls” appearance, it corresponds conceptually to what is now referred to as PCOM.

### Data Collection

Data were collected at the time of recruitment and during the follow-up period. At recruitment, data included age, BMI, obstetrical history, gynecological history (menarche, cycle regularity, amenorrhea, and oligomenorrhea), chronic illnesses (autoimmune, thyroid, psychiatric, malignant neoplasms, diabetes, dyslipidemia, neurologic problems, prolactinoma, respiratory, cardiovascular, and hypertension illnesses), clinical symptoms of hyperandrogenism (hirsutism, acne, and alopecia), and hormonal profile, including levels of luteinizing hormone (LH) and androgens, such as androstenedione, testosterone, dehydroepiandrosterone sulfate (DHEA-S), dehydroepiandrosterone (DHEA), 17-hydroxyprogesterone, and free testosterone. Androgens were measured using standard clinical assays (radioimmunoassay or enzyme-linked immunosorbent assay), and results were interpreted using contemporaneous laboratory-specific reference ranges (Table 1). Additional data included the metabolic panel (fasting insulin, glucose, and lipid levels and glucose tolerance tests) and sonographic appearance of ovaries. During the follow-up, data included age, death, and chronic conditions, such as diabetes (insulin-dependent or nondependent diabetes), cardiovascular diseases, dyslipidemia, need for anticoagulation therapy (venous thromboembolism or atrial fibrillation), embolic events, ischemic heart disease, arrhythmia, cardiovascular risk factors needing aspirin treatment, cerebrovascular accidents, sleep apnea, hypertension, autoimmune diseases (Crohn disease, psoriasis, and ulcerative colitis), hypothyroidism and hyperthyroidism, neuropathy, Parkinson disease, Alzheimer disease, multiple sclerosis, epilepsy, tremor, respiratory illnesses (asthma and chronic obstructive pulmonary disease), depression, schizophrenia and psychosis, bipolar disorder, various cancers (breast, ovarian, colon, thyroid, lung, melanoma, kidney, lymphoma, and leukemia), gastrointestinal diseases (gallstones, gastroesophageal reflux disease , colon polyps, and diverticulitis), osteoporosis, rheumatoid conditions (rheumatoid arthritis, gout, ankylosing spondylitis, osteoarthritis, fibromyalgia, scleroderma, arthritis not otherwise specified, and dermatomyositis), end-stage kidney disease (kidney transplant), Addison disease, and cirrhosis.

**Table 1.** Baseline Study Population Characteristics

Variable	Women with PCOS, mean (SD) (N = 340)		*P* value	Mean difference (95% CI)
	PCOM (n = 189)	Non-PCOM (n = 151)		
Age at recruitment, y	28.03 (5.98)	32.57 (9.21)	<.001	−4.54 (−6.25 to −2.83)
BMI	27.20 (5.74)	25.31 (6.31)	.004	1.88 (0.61 to 3.15)
Basal serum measures				
LH, mIU/mL	7.28 (8.60)	3.47 (4.39)	<.001	3.80 (2.39 to 5.22)
Androstenedione, ng/dL^a^	225.21 (107.74)	130.37 (73.64)	<.001	94.84 (75.36 to 114.04)
Total testosterone, ng/dL^a^	90.78 (37.18)	52.74 (76.66)	<.001	37.75 (25.36 to 50.14)
DHEA-S, µg/dL^a^	237.41 (163.7)	148.15 (107.41)	<.001	89.26 (58.89 to 82.59)
DHEA, ng/mL^a^	7233.57 (4386.87)	4839.69 (3662.93)	<.001	2393.89 (1517.09 to 3270.68)
Basal free serum testosterone, pmol/L^a^	11.76 (6.96)	6.03 (8.02)	<.001	5.72 (1.10 to 7.25)
Fasting insulin, μIU/mL	12.84 (9.83)	8.85 (6.33)	<.001	3.99 (2.25 to 5.72)
Fasting glucose, mg/dL	67.9 (34.6)	62.5 (42.3)	.19	5.4 (−2.7 to 13.5)
Total cholesterol, mg/dL	167.57 (63.32)	147.1 (82.24)	.01	20.08 (4.25 to 36.29)
Triglycerides, mg/dL	97.35 (72.57)	72.57 (61.06)	<.001	24.78 (9.73 to 38.94)
HDL, mg/dL	43.63 (18.15)	39 (22.39)	.04	4.63 (0.39 to 8.88)
Cholesterol ratio^b^	70.64 (51.76)	48.36 (51.84)	<.001	22.28 (11.15 to 33.40)
Apolipoprotein B, mg/dL	56.23 (32.81)	39.07 (35.27)	<.001	17.16 (9.87 to 24.44)

Abbreviations: BMI, body mass index (calculated as weight in kilograms divided by height in meters squared); DHEA, dehydroepiandrosterone; DHEA-S, dehydroepiandrosterone sulfate; HDL, high-density lipoprotein; LH, luteinizing hormone; PCOM, polycystic ovarian morphology; PCOS, polycystic ovary syndrome.

SI conversion factors: To convert androstenedione to nanomoles per liter, multiply by 0.0349; apolipoprotein B to grams per liter, multiply by 0.01; cholesterol and HDL to millimoles per liter, multiply by 0.0259; DHEA to nanomoles per liter, multiply by 3.47; DHEA-S to micromoles per liter, multiply by 0.027; glucose to millimoles per liter, multiply by 0.0555; insulin to picomoles per liter, multiply by 6.945; LH to international units per liter, multiply by 1.0; total testosterone to nanomoles per liter, multiply by 0.0347; triglycerides to millimoles per liter, multiply by 0.0113.

^a^ Reference ranges from the McGill University Health Centre Laboratory were 8.65 to 49.03 ng/dl for total testosterone, 1.9 to 12.0 pmol/L for free testosterone, 28.49 to 307.69 ng/dL for androstenedione, 46.42 to 320.04 µg/dL for DHEA-S, and 1038.31 to 4614.72 ng/mL for DHEA.

^b^ Cholesterol ratio = total cholesterol divided by HDL.

### Outcome Measures

Primary outcomes were long-term morbidity and mortality. Chronic morbidity was assessed based on the presence of the previously mentioned chronic conditions, both as groups and individually. Secondary outcomes included baseline characteristics at enrollment, including BMI, metabolic profile, hormonal profile, ovarian sonographic characteristics, and clinical features.

### Statistical Analysis

Statistical analyses were conducted using SPSS statistical software version 28.0 (IBM). Continuous variables are presented as mean (SD) and were compared using independent-sample *t* tests; categorical variables were compared using χ^2^ tests. Normality was assessed with the Kolmogorov-Smirnov test and visual inspection. Despite some nonnormal distributions, parametric tests were retained due to adequate sample size and robustness; nonparametric tests (eg, Mann-Whitney *U* test) were used in small subsamples (eg, age at death) and yielded similar or more conservative *P* values. Log transformation was not applied given that primary comparisons were group based and focused on clinical interpretability rather than modeling continuous factors.

Multivariable logistic regression was used to assess the association between ovarian morphology and long-term morbidity, adjusting for age; BMI; fasting insulin, total cholesterol, and triglyceride levels; and follow-up duration. All covariates were measured at baseline during the recruitment visit. Androgen levels were not included in multivariable models to avoid multicollinearity with PCOM status given that they are closely correlated with this phenotype. *P* values presented in the logistic regression analysis are adjusted for multiple comparisons using the false discovery rate method. A post hoc Bonferroni correction was applied to the primary outcome (non–insulin-dependent diabetes [NIDD]) to confirm robustness of the finding. Variables with less than 5% missing data were imputed using series means. All tests were 2-sided, with significance set at *P* < .05. Data were analyzed from January to April 2024.

## Results

Of 720 women with PCOS and follow-up data, 340 women had baseline transvaginal sonographic information and were included in the analysis. Among them, 189 women had PCOM (mean [SD] age at enrollment, 28.03 [5.98] years) and 151 women did not (non-PCOM group; mean [SD age at enrollment, 32.57 [9.21] years). Women with PCOM were younger at enrollment (*P* < .001).

### Baseline Characteristics

Baseline characteristics are summarized in Table 1. The PCOM group had a higher mean (SD) BMI of 27.20 (5.74) vs 25.31 (6.31) in the non-PCOM group (*P* = .004). Significant differences were also observed in hormonal profiles; the PCOM group had higher mean (SD) basal serum levels of LH (7.28 [8.60] mIU/mL vs 3.47 [4.39] mIU/mL; *P* < .001), androstenedione (225.21 [107.74] ng/dL vs 130.37 [73.64] ng/dL; *P* < .001), testosterone (90.78 [37.18] ng/dL vs 52.74 [76.66] ng/dL; *P* < .001), DHEA-S (237.41 [163.7] µg/dL vs 148.15 [107.41] µg/dL; *P* < .001), DHEA (7233.57 [4386.87] ng/mL vs 4839.69 [3662.93] ng/mL; *P* < .001), and free testosterone (11.76 [6.96] pmol/L vs 6.03 [8.02] pmol/L; *P* < .001), with a greater proportion of women in the PCOM than the non-PCOM group exceeding the reference range for total testosterone (108 women [57.1%] vs 37 women [24.5%]), free testosterone (78 women [43.4%] vs 32 women [21.2%]), androstenedione (106 women [56.1%] vs 45 women [29.8%]), DHEA-S (73 women [38.6%] vs 28 women [18.5%]), and DHEA (63 women [33.3%] vs 22 women [14.6%]) levels. (To convert androstenedione to nanomoles per liter, multiply by 0.0349; DHEA to nanomoles per liter, multiply by 3.47; DHEA-S to micromoles per liter, multiply by 0.027; LH to international units per liter, multiply by 1.0; total testosterone to nanomoles per liter, multiply by 0.0347.) Additionally, the PCOM group showed higher mean (SD) levels of fasting insulin (12.84 [9.83] μIU/mL vs 8.85 [6.33] μIU/mL; *P* < .001), total cholesterol (167.57 [63.32] mg/dL vs 147.1 (82.24) mg/dL; *P* = .01), triglycerides (97.35 [72.57] mg/dL vs 72.57 [61.06] mg/dL; *P* < .001), high-density lipoprotein (HDL; 43.63 [18.15] mg/dL vs 39.07 [35.27] mg/dL; *P* = .04), and apolipoprotein B (56.23 [32.81] mg/dL vs 39.07 [35.27] mg/dL; *P* < .001), as well as a higher mean (SD) cholesterol ratio (70.64 [51.76] vs 48.36 [51.84]; *P* < .001). (To convert apolipoprotein B to grams per liter, multiply by 0.01; cholesterol and HDL to millimoles per liter, multiply by 0.0259; insulin to picomoles per liter, multiply by 6.945; and triglycerides to millimoles per liter, multiply by 0.0113.)

### Chronic Morbidity and Mortality

The mean age at follow-up was significantly different between groups; women with PCOM had a mean (SD) age of 61.7 (6.4) years compared with 67.7 (10.5) years among women without PCOM (*P* < .001). The mean (SD) follow-up period was 33.7 (2.3) years for women with PCOM and 35.1 (4.9) years for women without PCOM (*P* < .001). There were 7 deaths (3.7%) in the PCOM group and 9 deaths (5.96%) in the non-PCOM group, which was not statistically significantly different (*P* = .32). The mean (SD) age of death was younger in the PCOM group (54.3 [11.5] years), but this was not statistically significantly different compared with the non-PCOM group (70.8 [13.6] years) (*P* = .08).

NIDD was more prevalent in women with PCOM (45 women [23.8%]) compared with women without PCOM (14 women [9.3%]; *P* < .001). Rates of hypertension were higher in the PCOM group (65 women [34.4%]) compared with the non-PCOM group (38 women [25.2%]), although this difference was not statistically significant (*P* = .06). No significant differences between groups were detected in rates of other medical conditions (Table 2).

**Table 2.** Chronic Morbidity and Mortality at End of Study

Variable	Women with PCOS, No. (%) (N = 340)		***P* value**
	PCOM (n = 189)	Non-PCOM (n = 151)	
Age at follow-up, mean (SD), y^a^	61.71 (6.42)	67.67 (10.46)	<.001
Time in study, mean (SD), y^a^	33.68 (2.34)	35.10 (4.94)	<.001
Deaths	7 (3.70)	9 (5.96)	.32
Age at death, mean (SD), y^a^	54.25 (11.52)	70.83 (13.64)	.08
IDD	9 (4.76)	5 (3.31)	.50
NIDD	45 (23.80)	14 (9.27)	<.001
Dyslipidemia	53 (28.04)	43 (28.47)	.93
Receiving anticoagulation therapy^b^	4 (2.11)	4 (2.65)	.74
Ischemic heart disease	7 (3.70)	6 (3.97)	.89
Arrhythmia	2 (1.05)	4 (3.31)	.26
CVD or risk requiring aspirin treatment	16 (8.46)	19 (12.58)	.21
Cerebral vascular accident	0	1 (0.66)	.26
Sleep apnea	2 (1.06)	2 (1.32)	.82
Hypertension	65 (34.39)	38 (25.16)	.06
Chron disease	1 (0.53)	0	.37
Psoriasis	2 (1.06)	1 (0.66)	.69
Hypothyroidism	32 (16.93)	37 (24.50)	.08
Hyperthyroidism	1 (0.53)	0	.37
Neurologic disease	15 (7.93)	10 (6.62)	.64
Neuropathy or neuralgia	8 (4.23)	7 (4.63)	.85
Parkinson disease	1 (0.53)	0	.37
Alzheimer disease	0	1 (0.66)	.26
Epilepsy or seizures	0	2 (1.32)	.11
Multiple sclerosis	3 (1.58)	0	.12
Respiratory disease	23 (12.16)	18 (11.92)	.94
Asthma or COPD	21 (11.11)	17 (11.25)	.96
Psychiatric disease total	41 (21.69)	34 (22.51)	.85
Depression	27 (14.28)	27 (17.88)	.36
Anxiety	8 (4.23)	4 (2.65)	.43
Schizophrenia or psychotic medications	9 (4.76)	9 (5.96)	.62
Malignant neoplasm total	8 (4.23)	12 (7.94)	.14
Breast carcinoma	7 (3.70)	7 (4.63)	.66
Ovarian carcinoma	0	2 (1.32)	.11
Thyroid carcinoma	0	1 (0.66)	.26
Lymphoma or melanoma	0	3 (1.98)	.05
Lung carcinoma	1 (0.53)	2 (1.32)	.43
Gastrointestinal disease total	43 (22.75)	30 (19.86)	.51
Cholelithiasis	2 (1.06)	0	.20
GERD	40 (21.16)	27 (17.88)	.45
Colon polyps	0	2 (1.32)	.11
IBS	0	1 (0.66)	.26
Chron disease	1 (0.53)	0	.37
Rheumatologic disease total	11 (5.82)	5 (3.31)	.27
Rheumatoid arthritis	1 (0.53)	0	.37
Gout	3 (1.59)	2 (1.32)	.84
Osteoarthritis	2 (1.06)	0	.20
Fibromyalgia	1 (0.53)	0	.37
Scleroderma	0	1 (0.66)	.26
Osteoporosis	9 (4.76)	8 (5.30)	.82
End-stage kidney disease	1 (0.53)	0	.37

Abbreviations: COPD, chronic obstructive pulmonary disease; CVD, cardiovascular disease; GERD, gastroesophageal reflux disease; IBS, irritable bowel syndrome; IDD, insulin-dependent diabetes; NIDD, non–insulin-dependent diabetes; PCOM, polycystic ovarian morphology; PCOS, polycystic ovary syndrome.

^a^ The mean difference was −5.96 years (95% CI, −7.86 to −4.06 years) for age at follow-up, −1.42 years (95% CI, −6.25 to −2.22 years) for time in study, and −16.58 years (95% CI, −35.77 to 2.60 years) for age at death.

^b^ Treated for venous thromboembolism or atrial fibrillation.

### Logistic Regression Analysis

Data are presented in Table 3. The multivariate analysis indicated a significantly higher risk of NIDD in women with PCOM, with an aOR of 2.92 (95% CI, 1.06-2.82; *P* = .02). However, when controlling for confounding factors, no significant differences were observed in other disease states, including hypertension (aOR, 1.46; 95% CI, 0.82-2.56; *P* = .18). Controlled factors included age and BMI at recruitment; fasting insulin, cholesterol, and triglyceride levels at recruitment; and the follow-up period.

**Table 3.** Logistic Regression Analysis of Chronic Morbidity and Mortality Among Women With PCOM

Variable	aOR vs women without PCOM (95% CI)^a^	*P* value
IDD	0.63 (0.17-2.26)	.48
NIDD	2.92 (1.06-2.82)	.02
Dyslipidemia	1.00 (0.57-1.75)	.99
Receiving anticoagulation therapy^b^	1.09 (0.25-5.78)	.91
Ischemic heart disease	1.04 (0.28-3.82)	.94
Arrhythmia	0.47 (0.07-2.94)	.42
CVD or risk requiring aspirin treatment	0.58 (0.25-1.34)	.20
Cerebral vascular accident	NA	NA
Sleep apnea	0.23 (0.02-2.03)	.18
Hypertension	1.46 (0.82-2.56)	.17
Chron disease	NA	
Psoriasis	0.77 (0.06-8.70)	.83
Hypothyroidism	0.55 (0.30-3.00)	.052
Hyperthyroidism	NA	
Neurologic disease	1.29 (0.49-3.38)	.60
Neuropathy or neuralgia	1.04 (0.31-3.46)	.94
Parkinson disease	NA	NA
Alzheimer disease	NA	NA
Epilepsy or seizures	NA	NA
Multiple sclerosis	NA	NA
Respiratory disease	1.00 (0.48-2.06)	.98
Asthma or COPD	0.91 (0.43-1.92)	.80
Psychiatric disease total	0.99 (0.55-1.78)	.98
Depression	0.75 (0.39-1.45)	.39
Anxiety	2.34 (0.60-9.08)	.21
Schizophrenia or psychotic medications	0.92 (0.32-2.66)	.88
Malignant neoplasm total	0.56 (0.19-1.69)	.31
Breast carcinoma	0.71 (0.19-2.60)	.61
Ovarian carcinoma	NA	NA
Thyroid carcinoma	NA	NA
Lymphoma or melanoma	NA	NA
Lung carcinoma	0.39 (0.01-8.60)	.55
Gastrointestinal disease total	1.11 (0.61-2.04)	.72
Cholelithiasis	NA	
GERD	1.09 (0.59-2.01)	.76
Colon polyps	NA	NA
IBS	NA	NA
Chron disease	NA	NA
Rheumatologic disease total	2.16 (0.64-7.30)	.21
Rheumatoid arthritis	NA	NA
Gout	2.84 (0.32-24.70)	.34
Osteoarthritis	NA	NA
Fibromyalgia	NA	NA
Scleroderma	NA	NA
Osteoporosis	1.62 (0.52-5.06)	.40
End-stage kidney disease	NA	NA

Abbreviations: aOR, adjusted odds ratio; COPD, chronic obstructive pulmonary disease; CVD, cardiovascular disease; GERD, gastroesophageal reflux disease; IBS, irritable bowel syndrome; IDD, insulin dependent diabetes; NA, not available (not calculable due to absence of cases in 1 group); NIDD, non–insulin-dependent diabetes; PCOM, polycystic ovarian morphology.

^a^ All models were adjusted for age at enrollment; body mass index (calculated as weight in kilograms divided by height in meters squared); fasting insulin, total cholesterol, and triglyceride levels; and follow-up duration.

^b^ Treated for venous thromboembolism or atrial fibrillation.

## Discussion

In this cohort study, women with PCOM exhibited more severe metabolic disturbances at a young age during recruitment than women without PCOM. At the older age at follow-up, women with PCOM had a significantly higher prevalence of NIDD. Despite these differences, rates of other chronic conditions, mortality, and mean age of death did not differ between PCOM and non-PCOM groups.

This study provides unique insight into long-term morbidity and mortality in PCOS, comparing women with and without PCOM. With 37 years of follow-up, this study is among the longest prospective studies in this field. At recruitment, the mean age was approximately 30 years; by follow-up, women were approximately 65 years old, allowing for long-term assessment.

At enrollment, women with PCOM showed more severe metabolic disturbances, including higher BMI and increased levels of serum androgens, fasting insulin, total cholesterol, and triglycerides. These findings align with those of previous studies. Mills et al^11^ reported higher androgen levels and insulin resistance in women with PCOM at approximately age 30 years, along with elevated total and free testosterone levels, LH levels, and LH–follicle-stimulating hormone ratios. Excess androgen production was attributed to increased ovarian stromal volume, which comprises theca cells.^9,10^ Similarly, Christ et al^13^ found that polycystic morphology with high antral follicle count correlated with higher testosterone and androstenedione levels and LH–follicle-stimulating hormone ratios, indicating more severe hyperandrogenism and reproductive dysfunction. The median age of women in the study was 27.5 years. Their study also found associations of stromal echogenicity and the stromal-to-ovarian area ratio with androgen markers, supporting the association between sonographic features and metabolic disturbances in PCOS.

Women with PCOS face increased and persistent risk of metabolic syndrome. Schmidt et al^14^ reported higher prevalence of hyperglycemia and hypertension in postmenopausal women with PCOS, indicating sustained metabolic risk into older age. Ehrmann et al^15^ similarly found high metabolic syndrome prevalence in women with PCOS, particularly women with higher BMI and insulin levels. Additionally, studies by Schmidt et al^14^ and Wild et al^16^ found higher cardiovascular risk factors in postmenopausal women with PCOS compared with control groups. Scicchitano et al^2^ also reported increased cardiovascular risk factors in women with PCOS, particularly during reproductive age.

To our knowledge, our study is the first to show long-term morbidity differences in PCOS by ovarian morphology. Later in life, women with PCOM had a significantly higher prevalence of NIDD, which may reflect the more severe metabolic disturbances observed at enrollment when the women were younger. Interestingly, women with PCOM also had higher HDL cholesterol levels at recruitment, typically considered cardioprotective. Schmidt et al^14^ similarly reported that HDL levels decreased over time but initially were cardioprotective in women with PCOS. This higher HDL level in women with PCOM may be associated with the lack of significant differences in cardiovascular disease rates between the groups despite the higher prevalence of other metabolic disturbances in the PCOM group. However, HDL alone is unlikely to account for this finding. The younger follow-up age and low number of cardiovascular events may have limited our ability to detect differences. HDL’s protective effect may also diminish in the context of insulin resistance and diabetes, which were more common in the PCOM group. Despite the increased risk of NIDD at follow-up, when women were older, we did not observe significant differences in the prevalence of other conditions typically associated with insulin resistance, such as insulin-dependent diabetes, hypertension, cardiovascular diseases, and certain cancers. This may suggest that PCOM does not confer additional cardiometabolic risk beyond that already associated with PCOS.

Several factors may explain this discrepancy. Although follow-up was extensive, the cohort may still be too young to reveal some outcomes. A longer follow-up period could potentially reveal more significant differences. Second, PCOM may not add risk beyond PCOS itself. Additionally, the sample size for certain outcomes may have been insufficient to detect significant differences; notably, significantly lower hypothyroidism rates in the PCOM group may reflect subtle endocrine differences between phenotypes and highlight the need for larger, multicenter studies.

Schmidt et al^14^ found no increase in overall mortality in women with PCOS. Wild et al^16^ similarly reported that all-cause and cardiovascular mortality in women with PCOS were comparable to those of women in the general population. In our cohort, the mortality rate was similar in the groups, and there was no statistically significant difference in the mean age of death between PCOM and non-PCOM groups. Cause of death data were unavailable for most individuals, and the small number of events limited further analysis. Notably, women with PCOM were younger at recruitment and had shorter follow-up, likely reducing observed morbidity and mortality. This may strengthen our findings given that we would typically expect lower morbidity and mortality in the younger PCOM group.

Recent publications have questioned the current classification of PCOS phenotypes. The 2012 National Institutes of Health workshop supported continued use of the Rotterdam criteria but emphasized the need to report phenotypes individually based on the presence or absence of hyperandrogenism, ovulatory dysfunction, and PCOM.^17^ Myers et al^18^ proposed shifting classification toward metabolic features, such as insulin resistance and androgen excess, rather than relying on morphology alone. Unfer et al^19^ similarly suggested that PCOS with hyperandrogenism represents a distinct metabolic subgroup, while nonhyperandrogenic forms may reflect a different etiology. These perspectives support our finding that PCOM was associated with adverse metabolic profiles and increased risk of type 2 diabetes. In contrast, Chan et al^20^ reported that women without hyperandrogenism had lower insulin resistance and more favorable reproductive outcomes, consistent with our observation of reduced NIDD in the non-PCOM group. Additionally, Engmann et al^21^ found that racial and ethnic background significantly influenced the expression of PCOS metabolic features, reinforcing the syndrome’s heterogeneity. Together, these studies highlight the importance of phenotype-specific risk stratification in PCOS.

While an adverse metabolic profile at a younger age can indicate a higher risk for developing chronic conditions, it does not necessarily determine disease status at an older age. Various factors, including lifestyle changes, medical interventions, and genetic predispositions, can influence the progression and manifestation of diseases over time. Early intervention and continuous monitoring can alter long-term outcomes in individuals with high metabolic risk.^22,23,24^ Thus, although women with PCOM had more severe metabolic disturbances at a young age, these may not directly translate to higher disease rates later. Nonetheless, they justify continued monitoring of weight, glucose tolerance, and lipid profiles to reduce long-term risk, in line with PCOS management guidelines.^7^

### Strengths and Limitations

The strengths of this study include its long follow-up duration and comprehensive data collection on a wide range of chronic conditions. To our knowledge, this is the first study to evaluate the association between ovarian morphology and long-term morbidity and mortality in women with PCOS.

However, the study also has limitations. The relatively small sample size, particularly for certain outcomes, may limit the power to detect significant differences. Additionally, the single-center design may limit the generalizability of findings to other populations. Differences in study design, such as the inclusion criteria and diagnostic methods for PCOS, may also contribute to variations in findings across studies.^1,6^ Another limitation is the absence of race and ethnicity data, which were not routinely documented at our institution during enrollment. This limits assessment of phenotype-specific differences across racial and ethnic subgroups, as prior studies (eg, Engmann et al^21^) have shown such variation in PCOS expression. Antral follicle count, ovarian volume, and stromal parameters were not consistently recorded, so we could not compare specific sonographic features in association with hyperandrogenism. It could be claimed that the statistically significant finding in NIDD was due to multiple analyses being performed; however, even when adjusting for multiple comparisons using the Bonferroni correction, this outcome remained significant.

## Conclusions

This cohort study confirmed that PCOM in young women with PCOS was associated with increased androgen levels and worse metabolic profiles at baseline. There was a notably higher prevalence of NIDD in women with PCOM later in life. The observation of a younger age at death in women with PCOM, although not statistically significant, warrants further investigation. These findings underscore the need for targeted metabolic monitoring and preventive strategies in women with PCOS and PCOM. Future research should focus on multicenter studies to further explore these associations and develop effective interventions to improve long-term health outcomes in women with PCOS.

## Notes

Supplement.
Data Sharing Statement
