# Supplementary material for: Polycystic Ovarian Morphology and Chronic Morbidity and Mortality in PCOS
Source: JAMA Netw Open. 2025 Oct 31;8(10):e2540818. doi: 10.1001/jamanetworkopen.2025.40818 (PMC12579343; doi:10.1001/jamanetworkopen.2025.40818)
Supplement: Supplement. — Data Sharing Statement [file jamanetwopen-e2540818-s001.pdf]

Data Sharing Statement

Kugelman. Polycystic Ovarian Morphology and Chronic Morbidity and Mortality in PCOS. JAMA Netw Open. Published online October 31, 2025. doi:10.1001/jamanetworkopen.2025.40818

Data
Data available: No
